# Comparison of the effectiveness of diode laser, fluoride varnish, and their combination in treatment of dentin hypersensitivity: a systematic review of randomized clinical trials

**DOI:** 10.3389/froh.2025.1550127

**Published:** 2025-06-05

**Authors:** Manijeh Mohammadian, Nima Jalouti, Mohsen Yazdanian, Elham Keykha, Samira Hajisadeghi

**Affiliations:** ^1^ Department of Dental Biomaterials, School of Dentistry, Alborz University of Medical Sciences, Karaj, Iran; ^2^ Department of Dental Biomaterials, School of Dentistry, Iran University of Medical Sciences, Tehran, Iran; ^3^ Private Clinic, Tehran, Iran; ^4^ Research Center for Prevention of Oral and Dental Diseases, Baqiyatallah University of Medical Sciences, Tehran, Iran

**Keywords:** dentin hypersensitivity, diode laser, fluoride varnish, systematic review, randomized clinical trial

## Abstract

**Background and aim:**

Dentin hypersensitivity (DH) has long been a challenging condition, with many treatment methods showing limited success. However, the emergence of laser therapy, particularly the significant potential of diode laser (DL) and sodium fluoride (NaF) varnish, has sparked new hope. This research is a significant step towards a more effective treatment for DH, aiming to evaluate the promising potential of DL in treating DH, both independently and in combination with fluoride varnish. By delving into this research, you are investing your time in understanding a crucial advancement in the field of dentistry.

**Methods:**

A comprehensive search was conducted across the PubMed, Scopus, and Web of Science databases, including studies published up until May 2024. Randomized clinical trials that assessed DH using a visual analog scale (VAS) score were included. Data on participant demographics, treatment types, and VAS scores were extracted by two reviewers. The risk of bias was assessed using the revised Cochrane risk-of-bias instrument for randomized trials (RoB-2).

**Result:**

Three studies met the inclusion criteria, comparing NaF varnish, DL, and their combination. Both DL and the combination of DL and NaF varnish were more effective than NaF varnish alone in reducing DH. The combined treatment showed marginally superior outcomes compared to DL alone. Significant reductions in DH were observed across all treatment groups, with the combination therapy demonstrating the most substantial and consistent improvement.

**Conclusion:**

Diode laser therapy, particularly when combined with NaF varnish, represents a promising treatment approach for DH, offering superior efficacy over NaF varnish alone. These findings suggest that combination therapy may provide longer-lasting relief, with implications for improving clinical outcomes in DH management.

## 1 Introduction

Dentin hypersensitivity (DH) is a common condition that affects approximately 20% of people. It is characterized by sharp, rapid, and temporary or prolonged pain in response to various triggers (1–4). These stimuli, including thermal (TS), evaporative (ES), tactile, osmotic, or chemical, typically do not elicit any response in healthy teeth (1). Significantly, DH cannot be attributed to other forms of dental pathology (1, 3). In addition, DH may be considered an adverse effect of dental bleaching procedures (2).

DH can be attributed to several contributing factors. Gingival recession is a key factor, as it exposes the cervical dentin and root surface, making them vulnerable to various stimuli. Aging can also increase dentin sensitivity, as the protective enamel layer may wear down over time. Dehiscence of the soft tissue, or the separation of the gingiva from the tooth surface, can also contribute to dentin exposure and increased sensitivity. Furthermore, overly aggressive tooth brushing has been identified as a potential cause of DH (5).

Several concepts have been proposed to clarify the underlying mechanisms of DH. These theories, including the transducer, gate control, direct receptor mechanism, and modulation theories, attempt to explain how and why DH occurs. However, the hydrodynamic hypothesis is widely accepted as the most plausible explanation for DH (3, 6).

Various assessment measures may be used to quantify the severity of pain related to DH. These include a descriptive scale that classifies the pain as mild, moderate, or severe, and a visual analog scale (VAS) that allows patients to assess the pain on a scale from 0 to 10 (7).

Desensitizing agents are commonly used in dental practice to manage DH. These agents typically contain compounds such as sodium fluoride (NaF), nanohydroxyapatite, amorphous calcium phosphate, calcium, and sodium monofluorophosphate (2). The effectiveness of fluoride-based compounds in alleviating DH has been well-established (8–10). Fluoride promotes the formation of calcium fluoride (CaF_₂_) crystals within the dentinal tubules, which reduces dentin permeability. These crystals are highly resistant to dissolution in saliva, thus temporarily occluding the tubules (11). However, despite the widespread use of 2% NaF varnish in clinical settings, the calcium deposits formed can be easily removed through routine brushing and the flow of saliva.

Laser therapy has emerged as an alternative method for treating DH. Clinical studies report significant variability in treatment outcomes, with success rates in the range of 5%–100% (12, 13). Many patients experience immediate relief from sensitivity after laser treatment, and the effectiveness of the therapy is influenced by various factors, including the wavelength, irradiation mode (continuous or pulsed), exposure time, and power output (14). Although the precise mechanism of action is not fully understood, it is widely believed that lasers alleviate sensitivity by sealing dentinal tubules through a process of melting and recrystallization of the dentin, which reduces fluid movement and nerve stimulation (15).

Several types of lasers, including neodymium-doped yttrium aluminum garnet (Nd:YAG), erbium-doped yttrium aluminum garnet (Er:YAG), CO₂ lasers, and diode lasers, have been investigated for DH treatment. Among these, diode lasers have shown considerable effectiveness, often achieving results comparable to or better than conventional methods. In addition, low-level laser therapy (LLLT) – also known as cold laser therapy or photobiomodulation therapy – has attracted attention for its non-invasive approach and minimal thermal effects (2, 4, 7, 16). LLLT typically uses wavelengths in the red (630–690 nm) and near-infrared (810–980 nm) spectrum. It stimulates odontoblasts, promoting the formation of tertiary dentin, narrowing of dentinal tubules, and modulation of inflammation and pain through cellular activation (17). The therapeutic effectiveness of LLLT is dose-dependent and usually requires multiple sessions spaced over time. The response to LLLT varies among individuals, with outcomes influenced by laser parameters, treatment frequency, and the underlying cause of hypersensitivity. Notably, lasers can also be combined with conventional desensitizing agents to enhance clinical outcomes, offering a synergistic approach to managing DH effectively (4).

In this regard, this systematic review aimed to evaluate and compare the effectiveness of NaF varnish, diode laser therapy (DL), and their combination in reducing DH.

## 2 Methods

### 2.1 Search strategy

Following the PRISMA criteria (9), two researchers conducted thorough searches of several electronic databases, including PubMed, Scopus, Web of Science, and Google Scholar, covering publications published up to May 2024. The search methodology used a combination of Medical Subject Headings (MeSH) terms and text-based keywords. Alongside the electronic database searches, the researchers manually reviewed the reference lists of the selected articles and related review papers and meta-analyses to identify any potentially relevant publications. Search terms include: [(“fluoride varnish” OR “NaF varnish” OR “sodium fluoride” OR “diode laser” OR “sodium monofluorophosphate”) AND “dentin hypersensitivity”] (Table 1).

**Table 1:** Search strategies and results of the search procedure.

Database	Search strategy	Results
PubMed	(((“dentin hypersensitivity”[Title/Abstract]) OR (“Dentin Sensitivity”[Mesh])) OR (“tooth sensitivity”[Title/Abstract])) AND (((“fluoride varnish”[Title/Abstract]) OR (“diode laser”[Title/Abstract])) OR (“Lasers, Semiconductor”[Mesh]))	148
WOS	((TS = (“dentin hypersensitivity”)) OR TS = (“Dentin Sensitivity”)) OR TS = (“tooth sensitivity”) AND ((TS = (“fluoride varnish”)) OR TS = (“diode laser”)) OR TS = (“Lasers, Semiconductor”)	126
Scopus	(TITLE-ABS-KEY(“dentin hypersensitivity”) OR TITLE-ABS-KEY(“Dentin Sensitivity”) OR TITLE-ABS-KEY(“tooth sensitivity”)) AND (TITLE-ABS-KEY(“fluoride varnish”) OR TITLE-ABS-KEY(“diode laser”) OR TITLE-ABS-KEY(“Lasers, Semiconductor”))	303

### 2.2 Inclusion criteria and study selection

Studies were included if they were clinical trials published in English, comparing NaF varnish, diode laser, or their combination for treating dentin DH, and used a VAS score. PICO criteria were

• Population: individuals with DH
• Intervention: sodium fluoride varnish, diode laser, or their combination
• Comparison: placebo or comparisons between the interventions
• Outcome: reduction in DH, measured by VAS score

Once duplicate publications were removed, the remaining papers were screened based on their titles and abstracts to exclude irrelevant themes or articles that did not match the inclusion requirements. Subsequently, one reviewer (A) conducted a thorough assessment of the whole texts of the remaining publications. Any uncertainties were addressed through discussion with a second reviewer (B), ensuring a rigorous and transparent selection process. When multiple publications were found from the same study, the most comprehensive and/or latest paper was considered for inclusion.

### 2.3 Data extraction

Two researchers (A and B) independently reviewed each potentially eligible article and extracted the relevant information. A data extraction template was developed specifically for this review. The extracted data included participant age, gender, type of treatment (fluoride varnish, diode laser, and their combination), VAS score (before each treatment, after each treatment, and after combined treatment), and history of received DH treatments.

### 2.4 Risk of bias assessment

**Figure 1:**
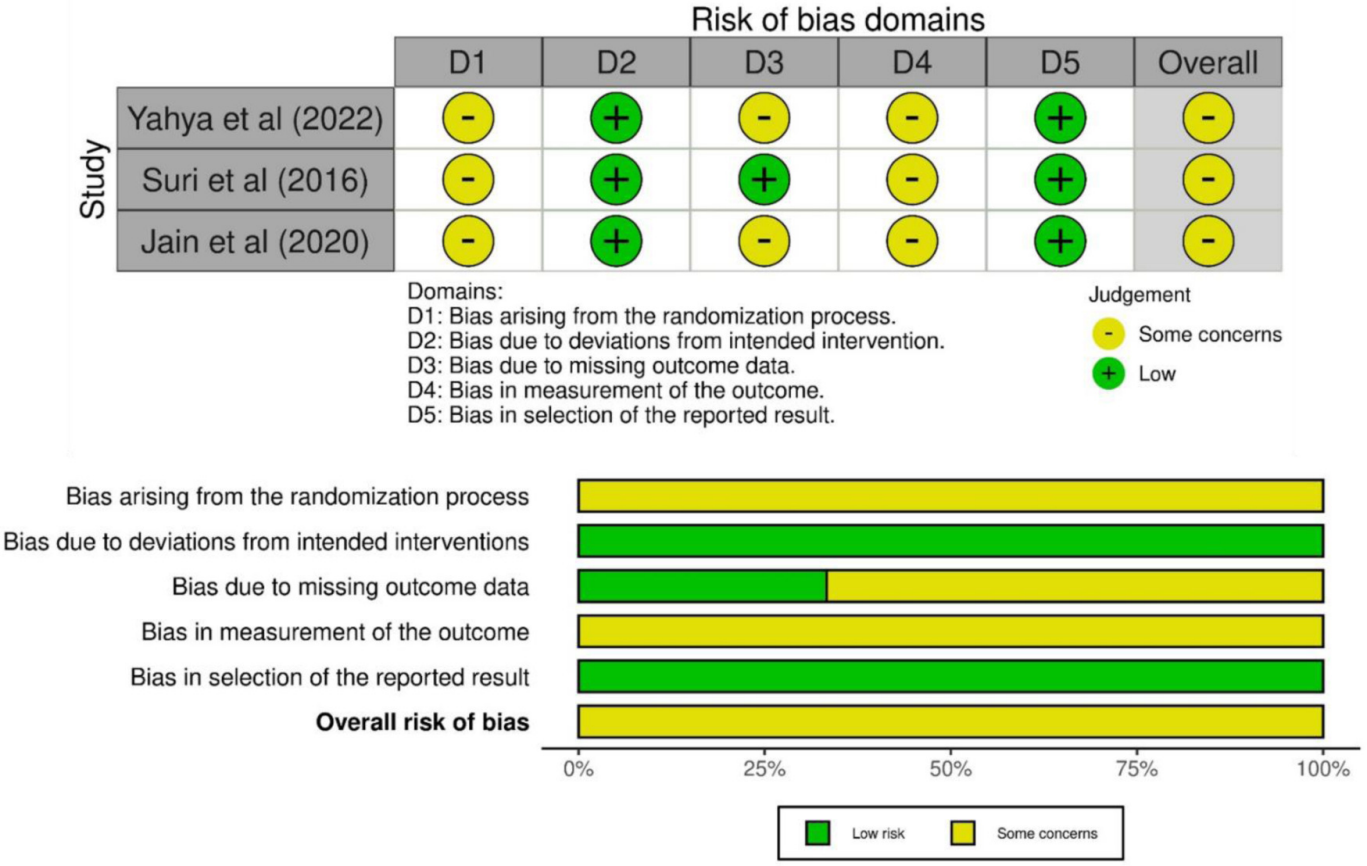
Risk of bias assessment (ROB 2.0).

The methodological quality of the included studies was assessed using the RoB-2 tool (Figure 1), which evaluates five domains: randomization, deviations from planned interventions, missing outcome data, outcome measurement, and selection of reported outcomes. Each domain was rated as “high,” “unclear,” or “low” risk of bias (18). All three studies clearly described randomization methods; however, allocation concealment was not adequately detailed, resulting in an unclear risk. Deviations from planned interventions were low. The risk of bias due to missing data was unclear due to insufficient reporting in two studies. Bias in outcome measurement was unclear in studies since none of them mentioned whether the outcome assessors were blinded to the intervention groups. The risk of selective reporting was low in all studies. Two separate reviewers undertook the quality assessment for all the papers in the review, referred to as reviewer A and reviewer B. Any discrepancies between the two reviewers' assessments were discussed to reach an agreement. In cases where the two reviewers could not resolve the disagreement, a third reviewer, reviewer C, was consulted to intervene and help determine the final quality rating for the disputed study.

## 3 Result

### 3.1 Study selection and characteristics

**Figure 2:**
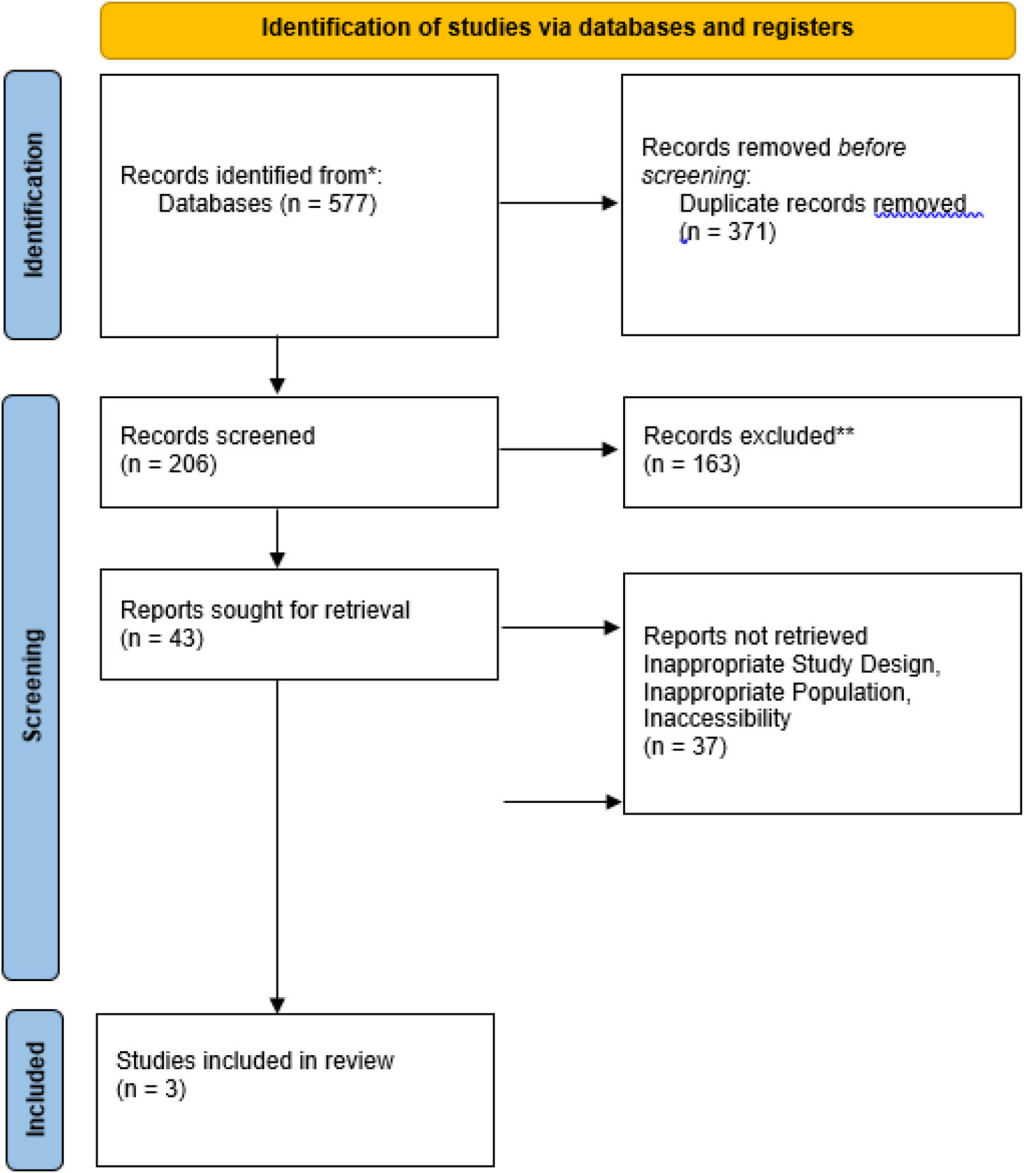
Flow diagram of study selection procedure.

The search strategy identified 577 studies across the selected databases (Figure 2). After removing 371 duplicates, 206 articles remained for title and abstract screening. Of these, 203 were excluded, resulting in the inclusion of three studies in the systematic review. Table 2 presents the characteristics and key findings of the included studies.

**Table 2:** Summary of characteristics of included studies.

Author	Year	Country	Study design	Randomization/allocation concealment	Group characteristics	Investigation parameters	Interventions	Follow-up duration	Dropouts	Results
Yahya et al. (2)	2022	Saudi Arabia	Interventional comparative study	Random allocation using table of random numbers; concealment not mentioned	39 dental students (13/group), aged 20–25 years, medically healthy, 136 teeth examined	Cold test and VAS scores pre- and post-bleaching	G1: 5% NaF varnish; G2: LLLT (660–900 nm); G3: NaF + LLLT; all post-bleaching	Immediately post-bleaching	None	All groups showed a significant reduction in sensitivity; no significant difference among groups (*p* = 0.544)
Suri et al. (6)	2016	India	Interventional study	Random allocation of teeth using table of random numbers; concealment not mentioned	30 patients (120 teeth); aged not mentioned; moderate to severe DH in canines and premolars; 1 sensitive tooth per quadrant	VAS scores with tactile and air-blast stimuli	G1: Placebo; G2: 5% NaF varnish; G3: 980 nm DL; G4: NaF + DL	Baseline, 24 h, 1 week, 1 month, 2 months	7	All interventions reduced DH; greatest reduction in G4 (NaF + DL) compared to G3 and G2
Jain et al. (3)	2020	India	Randomized split-mouth clinical trial	Random allocation using lottery method; concealment not mentioned	60 healthy adults aged 20–60 with DH in ≥3 quadrants; 626 teeth evaluated	VAS scores with air-blast, cold, and tactile stimuli	G1: 5% NaF varnish; G2: 810 nm DL (0.5W); G3: NaF + DL	1 week, 2 weeks, 1 month, 3 months, 6 months	None	Significant reduction in DH in all groups; G2 and G3 more effective than G1; G3 slightly better than G2, but not statistically significant

NaF, sodium fluoride; LLLT, low-level laser therapy; DH, dentin hypersensitivity; VAS, visual analog scale; DL, diode laser.

### 3.2 Demographic characteristics

A total of 150 participants were evaluated across the included studies. The study by Yahya et al. (2) involved 30 participants, though specific demographic details were not reported. Jain et al. (3) studied 60 patients, comprising 33 women and 27 men, with a mean age of 36 years. Baseline hypersensitivity scores did not differ significantly between groups (*p* ≥ 0.05). In the study by Suri et al. (6), 30 patients (120 teeth) were followed over a 2-month period. Although there was an equal gender distribution in the 40–49-year-old age group, there were more men than women in the other age categories (20–29, 30–39, and 50–59 years), this variation was not statistically significant. No adverse events were reported during the observation period in any study.

### 3.3 Intragroup changes in DH

All studies demonstrated significant reductions in DH within each group. In Yahya et al. (2), mean VAS scores rose from 4.80 ± 2.41 before bleaching to 6.00 ± 2.23 after bleaching, then declined to 3.72 ± 2.31 after treatment (*p* < 0.05). For the NaF varnish, DL, and combination groups, VAS scores decreased from 6.32 ± 2.21 to 3.89 ± 2.41, 5.83 ± 2.33 to 3.90 ± 2.38, and 5.83 ± 2.21 to 3.44 ± 2.16, respectively (*p* < 0.05 for all). Jain et al. (3) observed significant DH reductions at intervals of 1, 3, and 6 months across all groups (*p* ≤ 0.05) for air-blast, cold, and tactile stimuli, respectively. Similarly, Suri et al. (6) reported significant declines in tactile stimulation (TS) scores from baseline to 2 months: from 5.60 to 1.23 in the NaF group, 6.23 to 0.73 in the DL group, and 6.00 to 0.43 in the combination group. Air-blast scores also decreased significantly: from 6.70 to 1.80 in the NaF group, 6.30 to 1.27 in the DL group, and 6.27 to 0.87 in the combination group (*p* < 0.001 for all within-group changes).

### 3.4 Intergroup comparisons

Combination therapy consistently showed the greatest reduction in DH. In the study by Yahya et al. (2), mean post-treatment VAS scores were lowest in the combination group (3.44 ± 2.16) compared to the NaF (3.89 ± 2.41) and DL groups (3.90 ± 2.38), though not statistically significant (*p* = 0.544). Jain et al. (3) found significantly greater reductions in the DL and combination groups compared to the NaF group at all follow-ups (*p* < 0.05); however, differences between the DL and combination groups were not significant. Suri et al. (6) reported significant intergroup differences at multiple timepoints, with the combination group outperforming others as early as 24 h (*p* < 0.05), and at 1 week, 1 month, and 2 months (*p* < 0.001).

## 4 Discussion

This systematic review aimed to evaluate and compare the effectiveness of NaF varnish, DL, and their combination in reducing DH. Across the three included studies, all interventions demonstrated significant reductions in DH. These findings support the overall efficacy of NaF, DL, and their combined use in mitigating DH.

The mechanism of action for NaF varnish is attributed to the formation of calcium fluoride (CaF_₂_) crystals, which temporarily occlude the dentinal tubules. However, due to their small size (approximately 0.05 µm), these crystals are prone to dissolution or mechanical removal through brushing, salivary flow, and exposure to dietary acids, which can eventually reopen tubules and lead to a recurrence of symptoms. In contrast, diode laser therapy offers a potentially longer-lasting effect by inducing nerve desensitization and promoting internal obliteration of tubules through the stimulation of secondary dentin formation. This secondary dentin is less susceptible to mechanical wear, thereby extending the duration of desensitization (19).

All three studies affirmed the therapeutic effects of NaF and the diode laser, both individually and in combination. The reduction in sensitivity in the NaF-only groups may result from the interaction between fluoride and calcium ions in the dentinal fluid, forming a superficial layer of CaF₂ that partially blocks tubules.

The combined use of NaF and the diode laser showed promising outcomes in the studies by both Suri et al. and Jain et al., suggesting a synergistic effect between NaF's remineralizing capacity and the laser's biostimulatory properties. Previous studies have reported comparable findings, indicating that the diode laser, whether applied alone or in combination with fluoride varnish, demonstrated a significantly higher effectiveness compared to fluoride varnish alone (20–22). Similar findings were reported by Umberto et al. (23) and Kumar and Mehta (24), who observed a greater reduction in sensitivity scores (VAS and cold air-blast index) when both treatments were used together compared to either alone. The laser likely enhances desensitization by stimulating odontoblasts, promoting secondary dentin formation, and increasing pain thresholds via nerve depolarization at the dentin–pulp interface (25).

Despite these encouraging results, Yahya et al. (2) reported no significant differences between treatment groups immediately after bleaching, contrasting with the findings of Suri et al. (6) and Jain et al. (3), who demonstrated superior outcomes in the DL and combined therapy groups over time. This discrepancy may stem from differences in follow-up duration; Yahya et al. (2) conducted only an immediate post-treatment assessment, while Jain et al. (3) followed participants for up to 6 months, allowing for observation of longer-term effects.

The incremental benefit of combining NaF with DL, although evident in some studies, was not statistically significant in the study by Jain et al. (3), suggesting the possibility of a modest additive effect. Variability in laser parameters (e.g., wavelength and power), baseline DH severity, and application protocols may have influenced these results. For instance, Suri et al. (6) used a 980 nm DL at 2 W continuous wave (CW) – a setting supported by Liu et al. (26) for effective tubule sealing – while Jain et al. employed an 810 nm DL at 0.5 W CW and Yahya et al. (2) used a laser in the 660–900 nm range at 90 mW with no cooling. These methodological inconsistencies hinder direct comparisons and may contribute to the variation in outcomes.

Several other factors may account for discrepancies among the studies. First, all three studies used the VAS to assess pain, which is inherently subjective and highly dependent on individual pain thresholds (27). In addition, examiner-dependent factors – such as pressure applied during tactile testing, variability in air-blast force, and fluctuations in temperature during cold testing – could have contributed to result variability. A further limitation was the lack of placebo control in two studies (2, 3), which complicates interpretation of treatment-related effects relative to natural desensitization or placebo responses. Sample size limitations also warrant consideration. Although each study showed statistically significant findings, larger and more diverse samples would enhance the generalizability and statistical power of future research.

This study suggests that diode laser therapy, particularly when combined with 5% NaF varnish, may offer superior and longer-lasting relief from DH compared to either modality alone. These findings support the clinical utility of combination therapy for DH management. However, future clinical trials should aim for including placebo-controlled groups, standardizing laser parameters, and using objective pain assessment tools. Consistent follow-up intervals extending beyond 6 months are recommended to determine the longevity of therapeutic effects. In additionally, studies should consider controlling for confounding factors such as plaque levels and baseline oral hygiene. Larger multicenter trials would also be valuable to validate findings across broader populations and clinical settings.

## Data Availability

The original contributions presented in the study are included in the article/Supplementary Material, further inquiries can be directed to the corresponding author.
